# Supplementary material for: Prediction of a Multi-Gene Assay (Oncotype DX and Mammaprint) Recurrence Risk Group Using Machine Learning in Estrogen Receptor-Positive, HER2-Negative Breast Cancer—The BRAIN Study
Source: Cancers (Basel). 2024 Feb 13;16(4):774. doi: 10.3390/cancers16040774 (PMC10887075; doi:10.3390/cancers16040774)
Supplement: Supplementary file 1 [file cancers-16-00774-s001.zip › cancers-2707428-supplementary.pdf]

Table S1. The predictive result according to menopausal status or Ki-67 in each MGA cohort.

Data Cohort	Clinical variable	Modeling/Test	Accuracy	F1 Score
	**Menopausal status**			
MMP	Premenopausal	447/36	75.0%	0.8000
MMP	Postmenopausal	447/43	93.0%	0.9524
ODX	Premenopausal	1733/178	91.0%	0.9497
ODX	Postmenopausal	1733/128	83.6%	0.8955
Ensemble (MMP + ODX)	Premenopausal	2180/214	90.7%	0.9451
Ensemble (MMP + ODX)	Postmenopausal	2180/171	81.9%	0.8812
	**Ki-67 (%)**			
MMP	≥20	447/31	83.9%	0.7826
MMP	<20	447/48	85.4%	0.9176
ODX	≥20	1733/81	81.5%	0.8624
ODX	<20	1733/225	90.2%	0.9463
Ensemble (MMP + ODX)	≥20	2180/112	79.5%	0.8189
Ensemble (MMP + ODX)	<20	2180/273	89.7%	0.9438

MMP, Mammaprint; ODX, Oncotype DX.

Table S2. Subgroup analysis according to various clinical situations in Ensemble cohort.

Subgroup (Modeling/Test)	Sensitivity	Specificity	Precision	F1 Score	Accuracy
PR positive & Ki-67 <20 (2180/238)	0.9771	0.3000	0.9383	0.9573	0.920
PR positive & HG 1~2 (2180/308)	0.9449	0.5556	0.9414	0.9431	0.899
PR positive & premenopausal (2180/202)	0.9432	0.6923	0.9540	0.9486	0.911
Ki-67 <20 & HG 1~2 (2180/264)	0.9662	0.3704	0.9309	0.9482	0.905
Ki-67 <20 & premenopausal (2180/148)	0.9853	0.4167	0.9504	0.9675	0.939
HG 1~2 & premenopausal (2180/196)	0.9540	0.6818	0.9595	0.9568	0.923
PR positive & Ki-67 <20 & premenopausal (2180/139)	0.9846	0.4444	0.9624	0.9734	0.950
PR positive & HG 1~2 & premenopausal (2180/187)	0.9524	0.6842	0.9639	0.9581	0.925
PR positive & Ki-67 <20 & HG 1~2 (2180/230)	0.9764	0.3333	0.9452	0.9606	0.926
Ki-67 <20 & HG 1~2 & premenopausal (2180/140)	0.9847	0.5556	0.9699	0.9773	0.957

MGA, multi-gene assay; PR, progesterone receptor; HG, histologic grade.

Table S3. The comparison for variable between prediction success group and fail group.

Variables		Test group (n=385)		
		Prediction success (n=334)	Prediction fail (n=51)	*p*-value
Age (mean±SD)		50.97±9.26	54.86±9.95	0.006
BMI (mean±SD)		23.22±3.22	23.52±3.46	0.542
Menarche age (mean±SD)		14.26±1.57	14.76±1.56	0.055
Menopausal status				0.012
	Premenopausal	194 (58.1%)	20 (39.2%)	
	Postmenopausal	140 (41.9%)	31 (60.8%)	
Preoperative E2 (mean±SD)		101.43±129.92	84.94±134.46	0.439
Preoperative FSH (mean±SD)		33.13±33.51	48.02±37.87	0.008
Tumor size (mean±SD)		1.66±0.71	1.95±0.94	0.010
Multiple lesion				0.960
	No	263 (78.7%)	40 (78.4%)	
	Yes	71 (21.3%)	11 (21.6%)	
HG				0.021
	1	91 (27.2%)	7 (13.7%)	
	2	218 (65.3%)	35 (68.7%)	
	3	23 (6.9%)	9 (17.6%)	
	Unknown	2 (0.6%)	0 (0.0%)	
NG				0.041
	1	24 (7.2%)	0 (0.0%)	
	2	272 (81.4%)	40 (78.4%)	
	3	36 (10.8%)	11 (21.6%)	
	Unknown	2 (0.6%)	0 (0.0%)	
EIC				0.767
	No	190 (56.9%)	27 (52.9%)	
	Yes	101 (30.2%)	18 (35.3%)	
	Unknown	43 (12.9%)	6 (11.8%)	
LVI				0.848
	No	289 (86.5%)	44 (86.3%)	
	Yes	43 (12.9%)	7 (13.7%)	
	Unknown	2 (0.6%)	0 (0.0%)	
SLN (mean±SD)		2.75±2.20	2.90±1.36	0.626
Lymph node metastasis				0.009
	No	280 (83.8%)	35 (68.6%)	
	Yes	49 (14.7%)	16 (31.4%)	
	Unknown	5 (1.5%)	0 (0.0%)	
Perinodal extension				0.764
	No	304 (91.0%)	45 (88.3%)	
	Yes	8 (2.4%)	2 (3.9%)	
	Unknown	22 (6.6%)	4 (7.8%)	
Estrogen receptor				0.432
	Low (0~5)	12 (3.6%)	3 (5.9%)	
	High (6~8)	322 (96.4%)	48 (94.1%)	
Progesterone receptor				0.008
	Negative	44 (13.2%)	14 (27.5%)	
	Positive	290 (86.8%)	37 (72.5%)	
HER2 receptor				0.038
	0	96 (28.7%)	6 (11.8%)	
	1+	146 (43.8%)	28 (54.9%)	
	2+	92 (27.5%)	17 (33.3%)	
Ki-67 (mean±SD)		15.63±13.96	20.39±13.94	0.024
MGA risk group				<0.001
	Low risk	287 (85.9%)	27 (52.9%)	
	High risk	47 (14.1%)	24 (47.1%)	

BMI, body mass index; E2, estradiol; FSH, follicle-stimulating hormone; HG, histologic grade; NG, nuclear grade; EIC, extensive intraductal component; LVI, lympho-vascular invasion; SLN, sentinel lymph node; HER2, human epidermal growth factor receptor 2; MGA, multi-gene assay.
